# A Novel Joint Denoising Method for Hydrophone Signal Based on Improved SGMD and WT

**DOI:** 10.3390/s24041340

**Published:** 2024-02-19

**Authors:** Tianyu Xing, Xiaohao Wang, Kai Ni, Qian Zhou

**Affiliations:** Division of Advanced Manufacturing, Shenzhen International Graduate School, Tsinghua University, Shenzhen 518055, China

**Keywords:** improved symplectic geometry modal decomposition (ISGMD), wavelet threshold (WT), the energy contribution (EC), spectral clustering (SC), spectral entropy (SE), denoising

## Abstract

Underwater acoustic technology as an important means of exploring the oceans is receiving more attention. Denoising for underwater acoustic information in complex marine environments has become a hot research topic. In order to realize the hydrophone signal denoising, this paper proposes a joint denoising method based on improved symplectic geometry modal decomposition (ISGMD) and wavelet threshold (WT). Firstly, the energy contribution (EC) is introduced into the SGMD as an iterative termination condition, which efficiently improves the denoising capability of SGMD and generates a reasonable number of symplectic geometry components (SGCs). Then spectral clustering (SC) is used to accurately aggregate SGCs into information clusters mixed-clusters, and noise clusters. Spectrum entropy (SE) is used to distinguish clusters quickly. Finally, the mixed clusters achieve the signal denoising by wavelet threshold. The useful information is reconstructed to achieve the original signal denoising. In the simulation experiment, the denoising effect of different denoising algorithms in the time domain and frequency domain is compared, and SNR and RMSE are used as evaluation indexes. The results show that the proposed algorithm has better performance. In the experiment of hydrophone, the denoising ability of the proposed algorithm is also verified.

## 1. Introduction

Thanks to the abundant resources in the ocean, oceanic exploration has received widespread attention [1,2]. Underwater acoustic technology is the main means of obtaining information in the ocean due to its advantages in transmission range [3], propagation speed, and energy loss. Since accurate underwater acoustic signals play an important role in the detection and localization, underwater acoustic communication, and data transmission, hydrophones have become a research hotspot as devices for receiving underwater acoustic signals. Influenced by the complex marine environment including waves, marine organisms, and ships, hydrophone signals [4,5] inevitably contain noise, which poses a challenge for acquiring information in the ocean. Thus denoising the hydrophone signals to obtain useful signals has important significance.

The noise in underwater acoustic signals is usually strong random and time-varying in complex and variable marine environments, which brings huge challenges to the extraction of useful signals. Compared with improving hydrophone hardware structure, denoising algorithms are widely used in underwater acoustic signal processing. Some algorithms including Fourier transform, Kalman filter (KF), and wavelet transform (WT) have good denoising effects [6,7,8], but they inevitably have some inherent defects. The Fourier transform can reflect the relationship between the time function and the spectral function but cannot present the specific details in the signal [9], so it is not applicable to the processing and analysis of non-stationary signals. KF filters and predicts signals by matrix operations, which results in algorithms that are computationally intensive and cause signal distortion [10]. Wavelet transform, as the most common denoising algorithm, is widely used in the field of medical devices [11] and marine technology [12], thanks to its ability to well reflect the local characteristics of the signal in the time-frequency domain. The wavelet threshold denoising algorithm proposed by Donoho et al. [13] is usually used to analyze non-stationary signals and has excellent denoising ability, but using it alone for complete signals can cause signal distortion.

Empirical modal decomposition (EMD) was proposed by Hilbert Huang et al. in 1998 [14], as a novel adaptive decomposition method, which can deal with nonlinear and non-stationary time series, and is applied to the fields of fault diagnosis [15] and speech recognition [16]. EMD decomposes a signal based on its time-scale characteristics and breaks the signal into a finite number of intrinsic modal functions (IMFs) with order frequencies. There is some modal overlap between adjacent IMFs, inevitably leading to the aliasing noise present in the reconstructed signal. Wu [17] et al. proposed an Ensemble empirical modal decomposition (EEMD) method, which adds white noise to the signal modal decomposition process to eliminate modal aliasing. However, the instability and computational complexity have not been fully resolved in the algorithm. Yeh [18] et al. proposed a Complete Ensemble empirical modal decomposition (CEEMD) method, which adds auxiliary noise in the form of positive and negative pairs into the signal to reduce the number of noise sets and eliminate residual noise. CEEMD leads to stochastic signal decomposition results because each decomposition does not have a fixed number of intrinsic modal decompositions. Li [19] et al. proposed CEEMDAN combined with wavelet thresholding to process the underwater acoustic signal algorithm, which makes the underwater acoustic signal obtain a good denoising effect.

The variational modal decomposition (VMD) proposed by Zosso et al. in 2014, as an adaptive non-recursive signal processing method, overcomes modal aliasing by introducing a variational model [20]. Compared with EMD-based algorithms, VMD possesses strong robustness due to its solid theoretical support, which makes it widely used in engineering fields [21,22,23]. Regrettably, the VMD needs to be set with suitable parameters. An unreasonable number k for IMFs can result in under-decomposition or over-decomposition for the signal. The decomposition accuracy of the VMD [24] depends on the penalty factor α, which, on increasing, will make the bandwidth of the IMFs larger. Thus, a suitable parameter $\left[k,\;\alpha \right]$ is crucial for signal decomposition. Yan [25] et al. used the whale optimization algorithm to find the optimal solution for the VMD parameter. Then they calculated the correlation coefficients between the IMFs and the original signals, and finally realized the denoising of the hydrophone signals. Wang [26] et al. used a genetic algorithm to optimize the VMD and used wavelet thresholding for IMFs to complete the removal of noise from local discharge signals in cables. The optimal IMFS can be obtained by the combined MOPSO and VMD algorithm proposed by Zhou [27] et al. and time-frequency peak filtering (TFPF) is used in IMFs to achieve noise removal from the gyroscope signal.

Symplectic geometry spectral analysis (SGSA) is based on the symplectic geometry theory [28] and the components obtained by the symplectic geometry transformation keep the characteristics of the time series, which makes the series have a more accurate decomposition. SGSA can handle non-stationary signals well and possesses strong robustness, but manually setting the embedding dimension affects the signal decomposition results. In 2018, Pan [29] et al. proposed the symplectic geometry modal decomposition (SGMD) method, which adaptively obtains the embedding dimension by the power spectral density, and realizes denoising and fault diagnosis for rotating machinery. Chen et al. [30] introduced cyclic kurtosis and cyclic impact intensity into SGMD to accomplish the adaptive reconstruction of symplectic geometry components, and it effectively realizes denoising and fault diagnosis of gears. Yu et al. [31] combined SGMD with EMD to accomplish the denoising and debris feature extraction for inductive oil chip sensors. The combination of multiple algorithms to efficiently realize signal denoising is getting more attention.

In order to effectively remove the noise signals from the hydrophone signals, this paper conducts some exploratory research. This paper proposes a denoising algorithm that combines improved SGMD with wavelet thresholds. Specifically, the contributions in this paper are as follows:

1. This paper is the first to optimize the termination conditions in SGMD to improve its ability to remove noise signals.
2. For SGMD generating excessive symplectic geometry components (SGCs), this paper uses spectral clustering to categorize the SGC and efficiently aggregate the noisy signals.
3. The mixed signals are denoised by WT and the clusters are reconstructed to get the ideal denoised signal.

Section 2 introduces the algorithms and models used in this paper, including improved SGMD, spectral clustering, spectral entropy, and joint denoising algorithms. Section 3 illustrates the advantages of the proposed method in this paper by comparing the experimental results. Finally, Section 4 gives the conclusion.

## 2. Algorithms and Models

**Figure 1:**
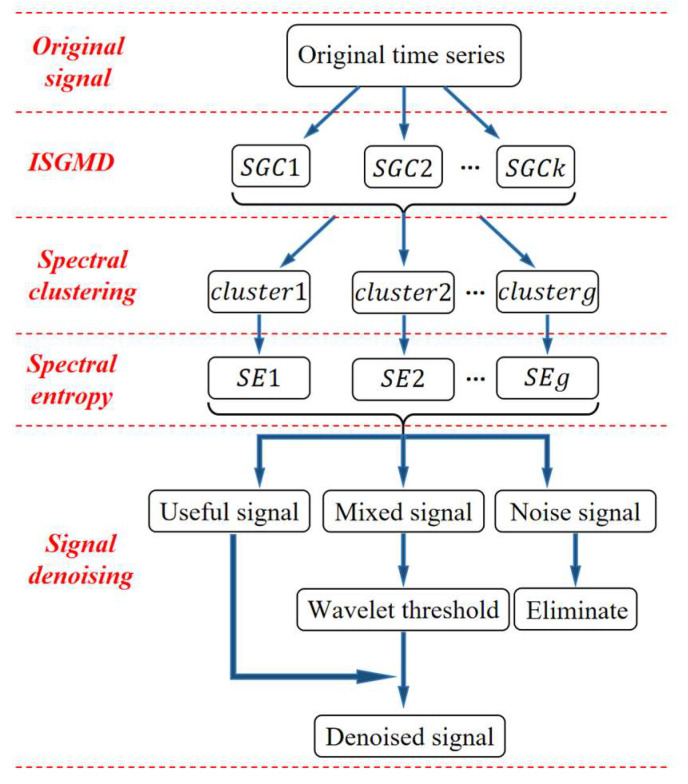
The framework of the algorithm combining many methods.

In signal denoising, a single denoising method makes it difficult to achieve the goal ideally, so the denoising algorithm combining many methods has become the main means. In order to realize the noise reduction for non-stationary signals and ensure the fidelity of the signals, this paper adopts the algorithm combining SGMD and WT to process the signals. For pre-denoising the signal during signal decomposition and obtaining better decomposition results, it introduces the energy contribution (EC) to improve SGMD. In addition, spectral clustering (SC) is used to cluster the decomposed signals according to signal structure features. Based on the idea of classification processing, spectral entropy (SE) is introduced to classify the clustered signals. Figure 1 shows the framework of the algorithm combining many methods.

### 2.1. Improved Symplectic Geometry Model Decomposition

**Figure 2:**
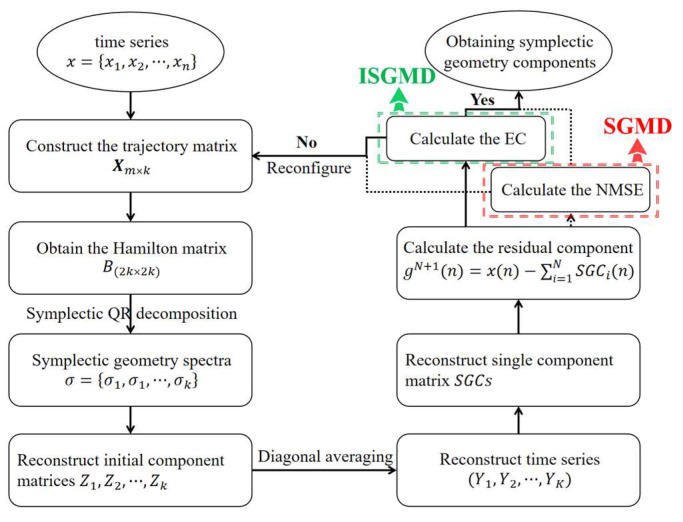
Flowchart of the SGMD and improved ISGMD.

SGMD solves the eigenvalues of the Hamiltonian matrix by the symplectic geometry similarity transform and uses the corresponding eigenvectors to reconstruct the symplectic geometry components (SGCs) [29]. SGMD uses a nonlinear transform and is suitable for the analysis of nonlinear signals. It can decompose a nonlinear signal and the decomposed sub-signal is nonlinear. The iteration termination condition in SGMD uses normalized mean square error (NMSE), which may cause overfitting of signal decomposition. Thus, this paper uses EC as the iteration termination condition to improve SGMD. The flowchart of the SGMD and improved SGMD (ISGMD) methods is shown in Figure 2.

#### 2.1.1. Symplectic Geometry Model Decomposition

1. Phase space reconstruction

$x=\left\{x_{1},x_{2},...,x_{n}\right\}$ denotes the original time series, where n represents the signal length. The trajectory matrix X is mapped by Taken’s embedding theorem [29].

(1) $$X=\left[\begin{array}{cc} \begin{array}{cc} x_{1} & x_{2}\\ x_{2} & x_{2+\tau } \end{array} & \begin{array}{cc} \cdots & x_{1+(k-1)\tau }\\ \cdots & x_{2+(k-1)\tau } \end{array}\\ \begin{array}{cc} \vdots & \vdots \\ x_{m} & x_{m+\tau } \end{array} & \begin{array}{cc} \ddots & \vdots \\ \cdots & x_{m+(k-1)\tau } \end{array} \end{array}\right]$$

In Equation (1), k is the embedding dimension and τ is the delay time, and m=n−(k−1)τ. The embedding dimension k and the delay time τ directly affect the trajectory matrix X. When τ=1, the trajectory matrix X is a Hankel matrix, and the elements of the matrix that are symmetric about the diagonal are the same [32]. The trajectory matrix X should remain a Hankel matrix, and the delay time in phase space should be 1. In this paper, the embedding dimension k is determined by using the power spectral density (PSD), and the frequency of the maximum peak $f_{max}$ is estimated by the PSD of the initial time series x. During the iteration, the embedding dimension k is set to k/3 if the normalization frequency is less than the given threshold $10^{-3}$. Otherwise, k is set to $k=1.2\times (f_{max}/f_{s})$, where $f_{s}$ represents the sampling frequency, and k takes an integer value less than k if not an integer value.

2. Symplectic geometry similarity transformation

The trajectory matrix X is analyzed by autocorrelation to obtain the covariance symmetry matrix $A=X^{T}X$, and a Hamilton matrix is constructed [33]:

(2) $$B=\left[\begin{array}{cc} A & 0\\ 0 & {-A}^{T} \end{array}\right]$$

According to Equation (2), set $C=B^{2}$, and both C and B are Hamiltonian matrices by the definition of Hamiltonian matrix. Thus, the symplectic orthogonal matrix Q can be constructed as Equation (3):

(3) $$Q^{T}CQ=\left[\begin{array}{cc} D & R\\ 0 & D^{T} \end{array}\right]$$

where the matrix Q is a symplectic matrix containing the orthogonality [34]. During the symplectic transformation, due to the structural features of the Hamiltonian matrix being maintained, the transformed matrix is a Hamiltonian matrix. D is the upper triangular matrix, i.e., $h_{ij}=0(i>j+1)$. The matrix C can transform to the matrix D by using the Schmidt transform and the eigenvalues of the matrix D can be obtained by the *QR* algorithm as Equation (4):

(4) $$\sigma (D)=\left\{\sigma_{1},\sigma_{2},\cdots ,\sigma_{k}\right\}$$

Assuming that A is a real symmetric matrix, the eigenvalues of A are the same as those of D, and the eigenvalues λ(X) of X are the square roots of σ(D):

(5) $$\lambda_{j}=\sqrt{\sigma_{j}}\;,\;j=1,2,\cdots ,k$$

Sorting the eigenvalues of A by order as Equation (5):

(6) $$\sigma_{1}>\sigma_{2}>\cdots >\sigma_{k}$$

The matrix Q represents the symplectic eigenvectors of A and $Q_{i}(i=1,2,\cdots ,k)$ are the eigenvectors corresponding to the eigenvalues $\sigma_{i}$ of the matrix A. Let $S=Q^{T}X,$ Z=QS, and Z is the reconstructed trajectory matrix. i-th row in the transformed coefficient matrix $S_{i}$ is denoted as Equation (7):

(7) $$S_{i}=Q_{i}^{T}X\;\;(i=1,2,\cdots ,k)$$

The corresponding reconstruction matrix can then be expressed as Equation (8):

(8) $$Z_{i}=Q_{i}S_{i}\;(i=1,2,\cdots ,k)$$

where $Z_{i}$ is the reconstructed single-component matrix. Thus, the reconstructed trajectory matrix in phase space is Equation (9) [29]:

(9) $$Z=Z_{1}+Z_{2}+\cdots +Z_{k}$$

3. Diagonal averaging

The reconstructed phase space matrix Z can be transformed by diagonal averaging to a sum of k symmetric geometric components with length n.

Define the dimension of the reconstructed trajectory matrix as m×k and reorder the time series of length n using the diagonal averaging technique. The original time series x is decomposed into d time series. Defining matrix $Z_{m*k}={\left(z_{ij}\right)}_{m*k}$, where 1≤i≤m*,* 1≤j≤k, $m^{*}=max(m,\;k)$ and m=n−(k−1)τ. If m<d, set $z_{ij}^{*}=z_{ij}$, otherwise, $z_{ij}^{*}=z_{ji}$. Then the elements $y_{t}(t=1,2,\cdots ,k)$ in $Y_{i}$ are transformed [35] as Equation (10):

(10) $$y_{t}=\left\{\begin{array}{c} \begin{array}{cc} \frac{1}{t}\sum_{k=1}^{t}y_{q,\;t-q+1}^{*} & \;\;\;\;\;\;\;\;\;\;\;\;\;\;\;\;\;\;\;\;\;\;\;1\leq t\leq k^{*} \end{array}\\ \begin{array}{cc} \frac{1}{k^{*}}\sum_{q=1}^{k^{*}}y_{q,\;t-q+1}^{*} & \;\;\;\;\;\;\;\;\;\;\;\;\;\;\;\;\;\;\;\;\;\;\;k^{*}<t<m^{*} \end{array}\\ \begin{array}{cccc} \frac{1}{n-t+1}\sum_{q=t-d^{*}+1}^{n-d^{*}+1}y_{q,\;t-q+1}^{*} & & & m^{*}\leq t\leq n \end{array} \end{array}\right.$$

According to the diagonal averaging formula, the matrix $Z_{i}$ is converted to a series $Y_{i}(y_{1},y_{2},\cdots ,y_{k})$ with the corresponding length.

4. Component reconstruction

The initial components are not completely independent, and there may be some relationship between them. Analyzed from a frequency perspective, component signals with similar frequencies can be considered to come from the same original signal. First, the first symplectic geometric component SGC1 is composed by $Y_{1}$ and other component signals containing a similar frequency to $Y_{1}$. Then SGC1 is removed from the initial signal and the remaining components are represented by $g^{1}$. However, the original signal usually contains noise signals, which can affect the signal reconstruction, so a termination condition is required for signal reconstruction. The NMSE between the residual signal and the original signal is usually set as the termination condition. The remaining signal will stop decomposition when the NMSE is less than the set threshold $h=10^{-2}$. The NMSE equation is as Equation (11):

(11) $${NMSE}^{h}=\frac{\sum_{e=1}^{n}g^{h}(e)}{\sum_{e=1}^{n}x(e)}$$

where h represents the number of iterations and $g^{h}$ represents the remaining signal. The initial signal is eventually decomposed into the sum of several component signals and the residual component signal $g^{N+1}$ as Equation (12):

(12) $$x(n)=\sum_{i=1}^{N}{SGC}_{i}(n)+g^{N+1}(n)$$

where N represents the number of decomposed component signals.

#### 2.1.2. Improved Component Reconstruction

The termination conditions in SGMD during the formation reorganization affect the number of SGCs and the ability of signal pre-denoising. NMSE, as the termination condition for SGMD, cannot deal with localized noise in the signal and may overfit the signal during denoising. For SGMD to achieve better signal pre-denoising, the energy contribution is used as the termination condition during the iteration. Energy contribution as the termination condition has many advantages [36,37], including a reasonable number of modes, better pre-denoising effect, taking into account both global and local features of the signal, and more adaptation to the signal structure.

The $u=\left\{u_{1},u_{2},\cdots ,u_{k}\right\}$ is denoted as a signal, where k represents the signal length. Then the total energy for the signal is calculated [38] as Equation (13):

(13) $$E\left[u\right]=\sum_{i=1}^{k}{\left|u_{i}\right|}^{2}$$

Setting the energy contribution as the termination condition, and the remaining signal will stop decomposition when the EC is less than the set threshold $Et=10^{-3}$. The energy contribution formula is as Equation (14):

(14) $${EC}^{h}=\frac{E\left[{g(e)}^{h}\right]}{E\left[u\right]}$$

where h represents the number of iterations and g(e) represents the remaining signal. The initial component signal is finally decomposed as $x(n)=\sum_{i=1}^{N}{SGC}_{i}(n)+g^{N+1}(n)$.

SGMD uses NMSE as an iterative termination condition, which focuses more on the similarity with the original signal, and the local features of the information cannot be represented. When the noise fluctuates violently, the decomposed signal also fluctuates. ISGMD uses the EC as an iterative termination condition, which can well extract the local features of the signal and can make the decomposition of the signal more stable.

### 2.2. Spectral Clustering

Spectral clustering can process the datasets and converge to a globally optimal solution by graph theory. A given dataset $A=\left\{a_{i}|a_{i}\in R^{d},\;i=1,2,\cdots ,k\right\}$, uses all the samples in graph A to construct a vertex set Y and set it into an undirected graph G=(Y,B,W) [39]. In a graph G, any two vertices can generate an edge, and all edges construct a set B. The element $w_{ij}$ in the affinity matrix W is called the affinity factor. The affinity factor indicates the similarity between $y_{i}$ and $y_{j}$, and the affinity factor $w_{ij}>0$. The element $w_{ij}$ in the affinity matrix W can be computed by using the similarity function [39]:

(15) $$w_{ij}=exp\left(\frac{-d^{2}(w_{i},\;w_{j})}{2\sigma^{2}}\right)$$

In Equation (15), d represents the Euclidean distance between two points, and the scale parameter σ>0.

Set $D=diag(d_{11},d_{22},\cdots ,d_{kk})$ to be the degree matrix and diag represents the diagonal matrix, where $d_{ii}=\sum_{j=1}^{k}w_{ij}$. L=D−W is the Laplacian matrix, and then computing the normalized graph Laplacian [40] as Equation (16):

(16) $$L_{ngm}=D^{-\frac{1}{2}}LD^{-\frac{1}{2}}=I-D^{-\frac{1}{2}}WD^{-\frac{1}{2}}$$

SGMD decomposition produces an excessive number of SGCs, and processing all of them consumes a large amount of computational resources. In order to quickly distinguish the information component from the noise component, the SGCs are processed by using the spectral clustering approach. The similarity metrics and graph structures on which SGMD and spectral clustering are based fit very well, which makes it easier to aggregate SGCs together based on similarity.

### 2.3. Spectral Entropy

The power spectrum of a signal is the cornerstone for spectral entropy [41], and spectral entropy is used to describe the irregularities of the signal spectrum. The spectral entropy utilizes the Fourier transform to calculate the energy distribution in the domain and combines it with the Shannon entropy to obtain the corresponding spectral entropy value [42]. The corresponding power spectrum of the signal is obtained by using the discrete Fourier transform [43]. At a certain frequency, the power spectrum possesses power $w_{i}$ and the probability of possessing power at this frequency is as Equation (17):

(17) $$p_{i}=\frac{w_{i}}{\sum_{i}w_{i}}$$

Let the length of the signal be k. The summation executes from i=1 to $i=\frac{k}{2}$ during using the discrete Fourier transform, and the power is normalized. The entropy of the power spectrum is denoted as Equation (18):

(18) $$H=-\sum_{i=f_{l}}^{f_{h}}p_{i}\log p_{i}$$

where $f_{l}$ and $f_{h}$ are the lower limit and upper limit, respectively. Normalizing the spectral entropy to get the normalized spectral entropy [43] as Equation (19):

(19) $$SE=\frac{H}{\log N_{f}}$$

where $N_{f}$ is the number of frequencies in $\left[f_{l},\;f_{h}\right]$.

Calculating the spectral entropy of a signal will help quickly distinguish the complexity of the signal. The more complex the signal, the greater the value of spectral entropy. By calculating the spectral entropy of a signal, the noise signal, mixed signal, and useful signal are quickly discriminated, which helps to denoise the signal more accurately.

### 2.4. Wavelet Threshold Denoising

The wavelet transform is the basis for wavelet thresholding. The mixed signal is decomposed by wavelet decomposition to produce different wavelet coefficients and the wavelet coefficients of the useful signals are larger than those of the noisy signals [44]. According to the theory, the mixed wavelet coefficients are processed by using a suitable threshold, and the wavelet coefficients of the useful signal are reconstructed to obtain the denoised signal. The steps of the wavelet threshold algorithm are as follows [45]:

#### 2.4.1. Wavelet Decomposition

The effect of signal decomposition relies on the choice of wavelet basis function, and a suitable wavelet basis function will give the ideal denoising effect. The characteristics of the signal need to be considered when the signal is processed by the wavelet threshold algorithm. The error in the signal and theoretical result is usually processed by wavelet analysis as a criterion for evaluating the quality of the wavelet basis function.

#### 2.4.2. Threshold for Wavelet Coefficients

The mixed wavelet coefficients are classified by a given threshold. The amplitude of the wavelet coefficients generated by the useful signal is larger than a given threshold, which needs to be reasonably preserved or reduced. The amplitude of the wavelet coefficients generated by the noise signal is smaller than a given threshold, which should be discarded. The choice of wavelet threshold affects the effectiveness of signal denoising. If the wavelet threshold is too large, useful information may be discarded; if the wavelet threshold is too small, noisy signals may be retained due to poor denoising.

#### 2.4.3. Wavelet Reconstruction

The denoised signal can be obtained by inverse wavelet transform on the processed wavelet coefficients. Compared to the noise signal, the useful signal has continuity in the time domain and the amplitude of the wavelet coefficients for the useful signal is greater than the amplitude of the wavelet coefficients for the noise signal in the wavelet domain, so the wavelet transform can separate the noise signal from the useful signal. If the wavelet threshold method is used for denoising, a reasonable threshold should be chosen. Methods of threshold selection include fixed threshold estimation, heuristic threshold estimation, unbiased likelihood estimation, etc. Usually, fixed threshold estimation and heuristic threshold estimation make it easier to remove useful signals from mixed signals. Thus, unbiased likelihood estimation is chosen as the threshold selection method. The steps are as follows [46]:

(1) Obtain the absolute value of each element in the signal y(j) and arrange them in ascending order to obtain a new signal sequence s(j) as Equation (20):
  (20) $$s(i)={\left(sort(|y|)\right)}^{2},\;\;\;\;(i=0,1,\cdots ,N-1)$$
(2) If the threshold (Th) uses the square root of the elements in the sequence s(j), the corresponding risk accompanying this threshold is risk(j) as Equations (21) and (22):
  (21) $$Th=\sqrt{s(j)},\;\;\;\;(j=0,1,\cdots ,N-1)$$
  (22) $$risk(j)=\frac{N-2j+\sum_{i=1}^{j}s(i)+(N-j)s(N-j)}{N}$$
(3) Find the point on the risk curve where the risky value is minimized and mark it as $j_{min}$. Thus, the minimum threshold is noted as Equation (23):
  (23) $$Th=\sqrt{s(j_{min})}$$

The uniform threshold is based on a Gaussian noise model as Equation (24):

(24) $$\lambda =\sigma \sqrt{2\ln N}$$

where N is the signal length and σ is the standard deviation for the noise. The standard deviation for the noise is usually estimated by using Equation (25):

(25) $$\sigma =\frac{median|v|}{0.6745}$$

where v is the wavelet coefficients and median represents the median function.

A threshold is the basis for the threshold function, and a suitable threshold function can ideally filter the mixed wavelet coefficients. Threshold functions are categorized into soft and hard threshold functions. They both can discard wavelet coefficients smaller than a given threshold as noise. When the wavelet coefficients are greater than a given threshold, the hard threshold function maintains the wavelet coefficients, while the soft threshold function subtracts the threshold from the original value. The soft threshold function makes the wavelet coefficients have better continuity, so in this paper the soft threshold function is chosen.

The hard threshold function [47] is denoted as Equation (26):

(26) $$\lambda (v,Th)=\left\{\begin{array}{cc} \begin{array}{c} v,\\ 0, \end{array} & \begin{array}{c} |v|\geq Th\\ |v|<Th \end{array} \end{array}\right.$$

The soft threshold function [47] is denoted as Equation (27):

(27) $$\lambda (v,Th)=\left\{\begin{array}{cc} \begin{array}{c} sign(v)(|v|-Th),\\ 0, \end{array} & \begin{array}{c} |v|\geq Th\\ |v|<Th \end{array} \end{array}\right.$$

Using wavelet thresholding for mixed signals further removes the noise signals from the mixed signals, which enhances the extraction of useful signals.

### 2.5. The Proposed Joint Denoising Algorithm (ISGMD-WT)

In the output signal of a hydrophone, the noise signal often swamps the useful signal. Keeping more useful signals while reducing noise is the pursued aim. This paper proposes a novel denoising algorithm combining ISGMD, SC, SE, and WT. The algorithm steps are as follows:

Step 1: Optimize the SGMD

The SGMD algorithm has a significant advantage for the decomposition of non-stationary signals and pre-noise reduction is accomplished during decomposition. SGMD using NMSE as the termination condition for decomposition hardly obtains an ideal pre-noising effect. Thus, SGMD uses energy contribution to optimize the termination condition in this paper. The ISGMD flowchart is shown in Figure 2.

Step 2: Symplectic geometry model decomposition

The hydrophone signals were decomposed by ISGMD to obtain a series of SGCs. The SGCs contain both useful and noise signals. The number of SGCs produced by SGMD is usually high, so this paper introduces spectral clustering to categorize SGCs into clusters.

Step 3: Cluster

To reduce the number of decomposed signals and quickly distinguish useful SGCs, the spectral clustering algorithm is adopted to cluster the SGCs. Spectral clustering is able to handle nonlinear data structures and aggregate noisy signals into the same cluster. In order to denoise purposely, this paper introduces spectral entropy to classify the aggregated clusters.

Step 4: Calculate and classify

In order to purposely remove the noisy signals, this paper uses spectral entropy to partition the clusters. The spectral entropy measures the randomness and complexity of the signal. By calculating the spectral entropy, clusters can be categorized into noise clusters, mixed clusters, and information clusters. Noisy signals are mainly concentrated in noise clusters, while useful signals are mainly concentrated in information clusters. Mixed clusters contain many noise signals and useful signals.

Step 5: Denoise and Reconstruct

Clusters are categorized into noise clusters, mixed clusters, and information clusters by spectral entropy. The noise clusters are directly discarded and the noise-free clusters are obtained by using wavelet thresholding to denoise the mixed clusters. The noise-free clusters and information clusters are reconstructed to obtain the denoised signal.

## 3. Simulation and Application

In order to verify the effectiveness and feasibility of the improved SGMD and wavelet threshold (ISGMD-WT) algorithm proposed in denoising hydrophone signals, two analog signals and one hydrophone signal are processed and analyzed in this paper. Signals in the ocean are complex and variable, so the two analog signals simulate a variety of underwater noise environments. The analog signal 1 is to explore the denoising capability of the ISGMD-WT algorithm in an environment containing signals of different frequencies and white noise. Analog signal 2 is to explore the denoising ability of the ISGMD-WT algorithm in an environment with different white noise intensities. To illustrate the advantages of the proposed algorithm in signal denoising, in the experiments, it is compared with the eminent signal denoising algorithms including the wavelet threshold (WT) algorithm, empirical modal decomposition and wavelet threshold (EMD-WT) algorithm, and the variational modal decomposition and wavelet threshold (VMD-WT) algorithm. It is displayed in the experimental results intuitively by using a signal-to-noise ratio (SNR) and root mean square error (RMSE) as evaluation indexes.

### 3.1. Simulation 1

**Figure 3:**
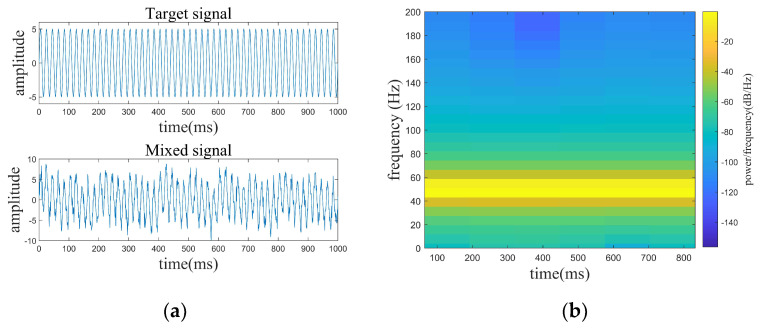
Target and mixed signals, spectrogram of the target signal. (**a**) Target and mixed signals. (**b**) Spectrogram of the target signal.

**Figure 4:**
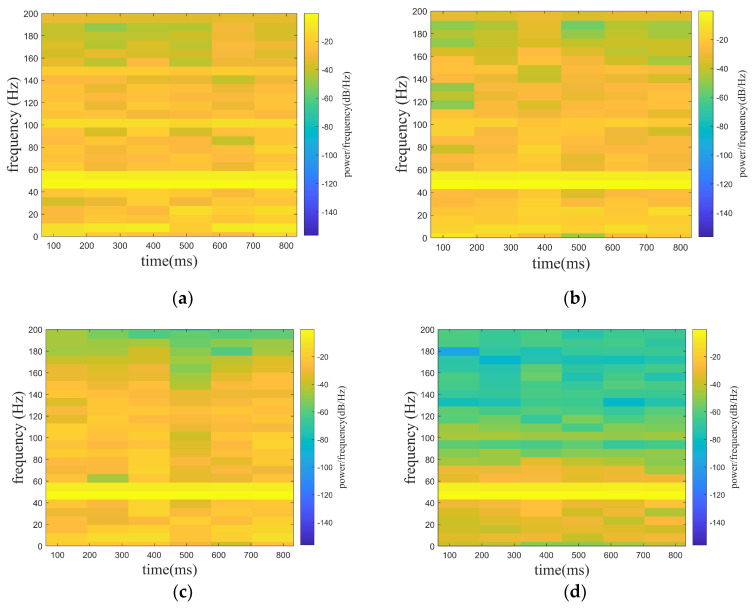
Denoising results of the four algorithms. (**a**) WT denoising algorithm. (**b**) EMD-WT denoising algorithm. (**c**) VMD-WT denoising algorithm. (**d**) ISGMD-WT denoising algorithm.

There are many different frequencies of noise in the ocean, and denoising the mixed noise containing different frequencies is an essential operation to extract the target signal. In this simulation experiment, the target signal is mixed with noise signals of other frequencies and Gaussian white noise is added. In the noise, the line spectrum signal of different frequencies represents the ship noise and the underwater acoustic communication signals during operation, and the white noise comes from marine life and wind waves. The mixed signals are as follows:

(28) $$\left\{\begin{array}{c} \begin{array}{c} f_{1}=5\times \sin\left(2\pi \times 50t\right)\\ f_{2}=\sin\left(2\pi \times 5t\right)\\ f_{3}=2\times \sin\left(2\pi \times 10t\right) \end{array}\\ \begin{array}{c} f_{4}=0.7\times \sin\left(2\pi \times 20t\right)\\ f_{5}=0.5\times \sin\left(2\pi \times 100t\right)\\ \begin{array}{c} f_{6}=WGN(t)\\ f=f_{1}+f_{2}+f_{3}+f_{4}+f_{5}+f_{6} \end{array} \end{array} \end{array}\right.$$

where the target signal $f_{1}$ has a frequency of 50 Hz, $f_{2}$, $f_{3}$, $f_{4}$, $f_{5}$, and $f_{6}$ are line spectrum signals with different frequencies. The target and mixed signals are displayed in Figure 3. The denoising results of the four denoising algorithms are shown in Figure 4. The denoising evaluation indexes of the four algorithms are recorded in Table 1.

From Figure 4, it can be known that all four algorithms can realize the denoising for the signal. However, the signals obtained by denoising using the WT, EMD-WT, and VMD-WT algorithms still contain significant noise, and the denoised signals possess fluctuations on the whole. Combined with the data in Table 1, the outstanding advantages of the ISGMD-WT in signal denoising can be clearly seen. The ISGMD-WT algorithm ensures the waveform smoothness during signal denoising, and the waveform remains highly consistent with the target signal. The proposed algorithm is significantly superior to the comparison algorithm in terms of SNR and RMSE.

### 3.2. Simulation 2

**Figure 5:**
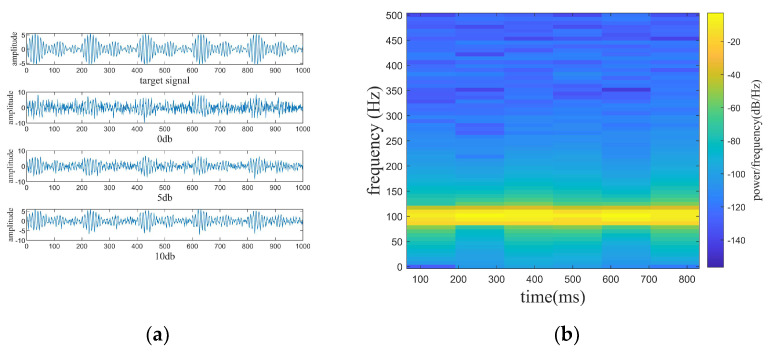
The original signal and the mixed signals with different noise decibels.

**Figure 6:**
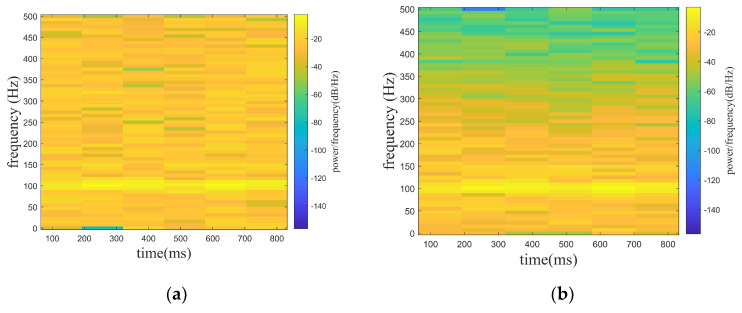

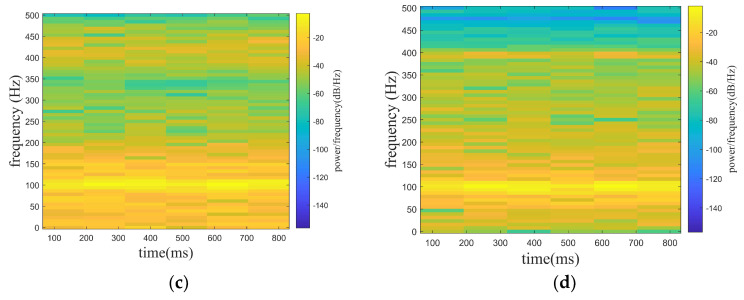
Denoising effect of four algorithms with 0 db noise decibel. (**a**) WT. (**b**) EMD-WT. (**c**) VMD-WT. (**d**) ISGMD-WT.

**Figure 7:**
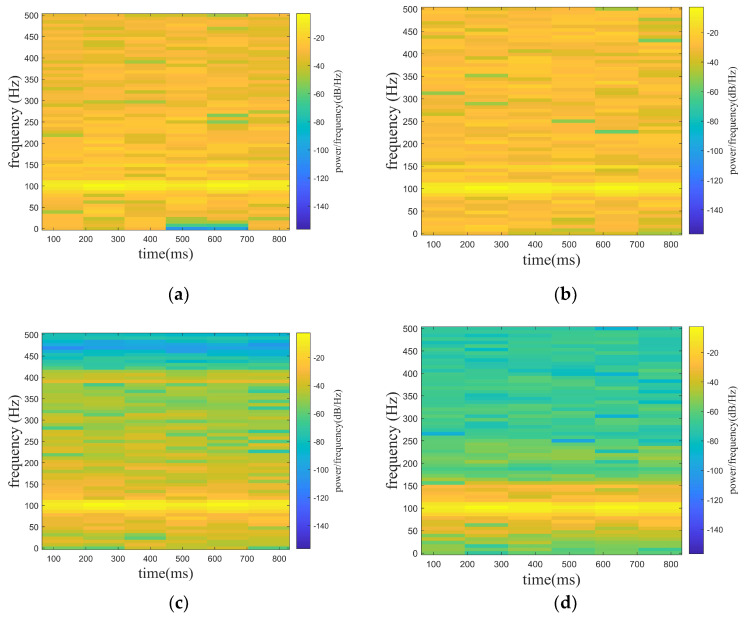
Denoising effect of four algorithms with 5 db noise decibel. (**a**) WT. (**b**) EMD-WT. (**c**) VMD-WT. (**d**) ISGMD-WT.

**Figure 8:**
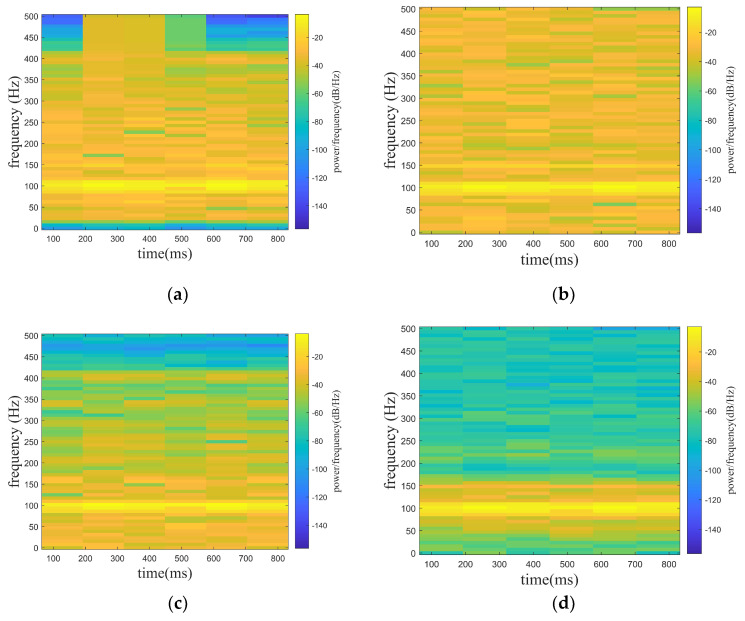
Denoising effect of four algorithms with 10 db noise decibel. (**a**) WT. (**b**) EMD-WT. (**c**) VMD-WT. (**d**) ISGMD-WT.

The complex and diverse noise in the ocean leads to the possibility that hydrophones may receive underwater acoustic signals that contain noise of different intensities. In the noise, the line spectrum signal represents the signal during the vehicle operation, and white signals of different intensities come from marine life and the variable marine environment. To simulate a mixed signal containing variable noise intensity, the simulated signal is set as follows:

(29) $$\left\{\begin{array}{c} \begin{array}{c} f_{1}=2\times \cos\left(2\pi \times 100t)[\sin(2\pi \times 5t)+\sin(2\pi \times 10t)+1\right]\\ f_{2}=0.5\times \cos\left(2\pi \times 150t\right) \end{array}\\ \begin{array}{c} f_{3}=A\times WGN(t)\\ f=f_{1}+f_{2}+f_{2} \end{array} \end{array}\right.$$

where $f_{1}$ is the target signal and $f_{2}$ is the line spectrum signal with a frequency of 150 hz. $f_{3}$ is a noise signal, and the A is set to be adjustable in order to satisfy the demand of different noise levels. f is the mixed signal (original signal) received by the hydrophone. In the paper, the noise signals are set with Gaussian white noise of 0 db, 5 db, and 10 db, where the db of white noise is the ratio of the target signal ($f_{1}$) and the line spectrum signal ($f_{2}$) to the white noise ($f_{3}$), while the SNR of the original signal is the ratio of the target signal ($f_{1}$) to the white noise ($f_{3}$). In Figure 5, Figure 5a shows the mixed signal with different noised decibels in the time domain, and Figure 5b shows the spectrogram of the target signal. Figure 6, Figure 7 and Figure 8 show the spectrograms of the denoising signals denoised by the four algorithms. Table 2 records the denoising evaluation indexes of the four algorithms under different noise intensities.

From Figure 6, Figure 7 and Figure 8 and Table 2, it can be intuitively seen that compared with the other algorithms, the proposed algorithm possesses eminent denoising ability and the denoised signal is highly consistent with the target signal. The algorithm proposed possesses high signal fidelity in the 0 db and 5 db noise decibel environments. In an environment with 10 db noise, the proposed algorithm essentially obtains the target signal from the mixed signal. It is clear that the denoising ability of the proposed algorithm in this paper is better than other algorithms.

### 3.3. Application in Hydrophone Experiment

**Figure 9:**
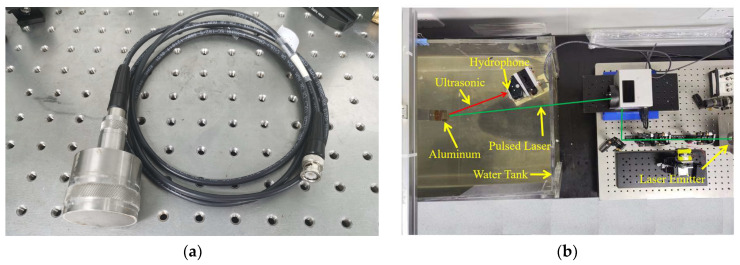
Hydrophone experiment. (**a**) The Olympus-v389-su hydrophone. (**b**) Experimental platform.

In order to verify the effectiveness of the algorithm in real signals, it is applied to the processing of hydrophone signals. The signals in this paper are received from the Olympus-v389-su hydrophone, which has a center frequency of 500 khz and a sampling frequency of 2 Mhz for collecting underwater acoustic signals. The hydrophone is shown in Figure 9a. The experimental platform is shown in Figure 9b. When the pulsed laser is irradiated on the aluminum block, the surface of the aluminum block breaks and emits ultrasonic waves. The ultrasonic waves are picked up by the hydrophone and used as an input signal for the experiment.

**Figure 10:**
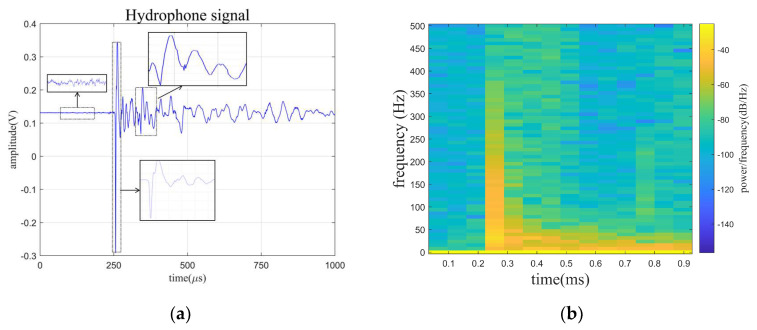
The time-domain signal received by the hydrophone and the spectrogram of signal. (**a**) Time-domain signal. (**b**) The spectrogram of signal.

**Figure 11:**
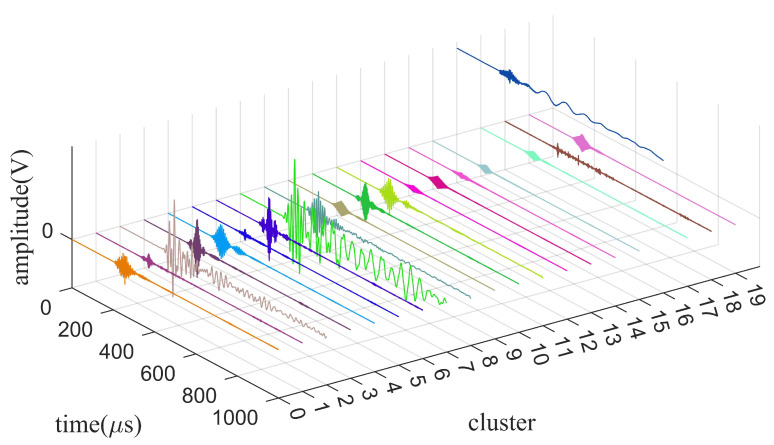
The clusters generated by spectral clustering.

Figure 10 shows the time-domain signal received by the hydrophone and the spectrogram of the signal. The hydrophone signal is divided into two main parts: the static phase, and the signal reception phase. The received signal in the static phase consists entirely of noise, and the signal in the signal reception phase consists of the noise and useful signals. In this paper, hydrophone signals are decomposed by the ISGMD algorithm and SGCs are clustered by using spectral clustering. The clusters are computed using spectral entropy, discarding the noise clusters and denoising the mixed clusters by wavelet thresholding to finally obtain the denoised signal. A time-domain diagram of the clusters is presented in Figure 11, which visualizes the waveform and amplitude of each cluster.

**Figure 12:**
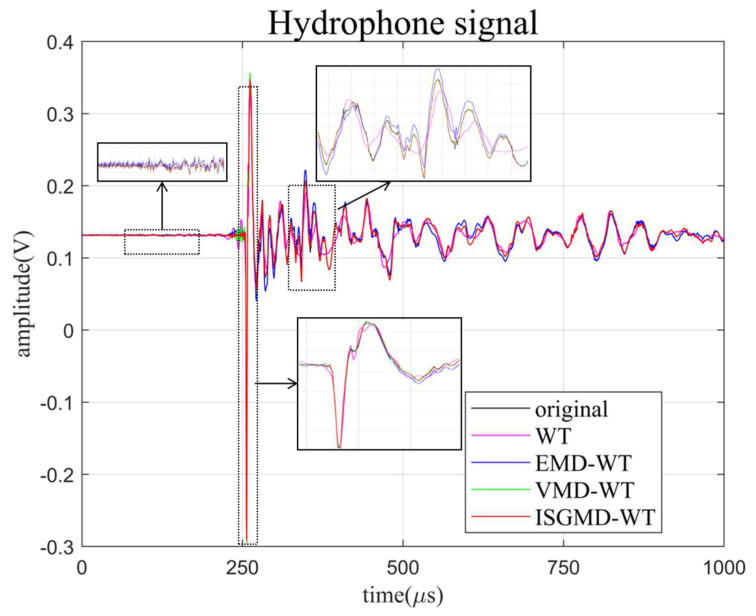
Denoised signals generated by the four algorithms.

**Figure 13:**
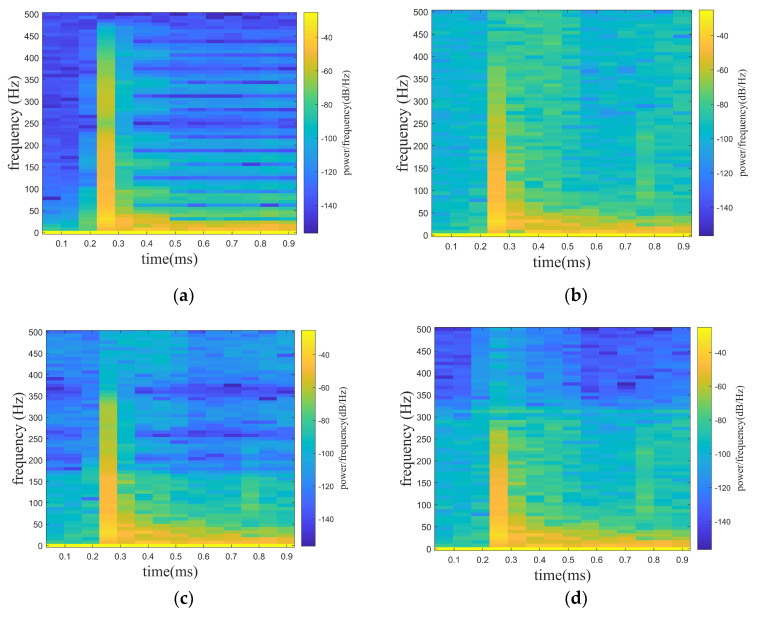
The spectrogram of denoised signals generated by the four algorithms. (**a**) WT. (**b**) EMD-WT. (**c**) VMD-WT. (**d**) ISGMD-WT.

The clusters generated by ISGMD and spectral clustering are shown in Figure 9. The ISGMD decomposes the signal into 51 SGCs, which are divided into 20 clusters by spectral clustering, and the clusters are computed using spectral entropy. The noisy signal is mainly in cluster 18, for which the noise-reduction process using wavelet thresholding is used to obtain the noise-reduced cluster. Signal reconstruction obtains the denoised hydrophone signal. The denoising signals generated by the four algorithms are shown in Figure 12 and Figure 13 and the evaluation indexes are recorded in Table 3. Figure 12 shows the time-domain waveform graph of the four algorithms after denoising the hydrophone signal, and Figure 13 illustrates the spectrograms of the four algorithms after denoising the hydrophone signal.

Combined with Figure 12 and Figure 13, and Table 3, the outstanding performance of the algorithm proposed in this paper in signal denoising can be clearly seen. In Figure 12, the ISGMD-WT algorithm can smooth the curve of the signal when the signal is denoised, especially in the face of sudden signal changes, and it can prevent the signal from distortion during noise reduction. Based on the data in Table 3, it is known that the ISGMD-WT algorithm improves the SNR and minimizes RMSE compared to the other algorithms. Combining the results of the above three experiments, the algorithm proposed in this paper can effectively denoise noises of different frequencies and intensities, and prevent modal aliasing during the denoising process. It can provide new ideas for the denoising of underwater acoustic signals, and can also be expanded to other areas of signal denoising, providing technical support for the application of signal denoising in engineering.

## 4. Conclusions

In order to overcome the noise in the hydrophone that affects signal availability, a joint denoising algorithm based on SGMD and WT is proposed in this paper. This paper is the first to introduce the energy contribution into SGMD as the iterative condition, which reasonably enhances the denoising ability of SGMD. Aiming at generating too many SGCs during SGMD, this paper uses spectral clustering to cluster the SGCs, effectively aggregating the noise signals into the same cluster. Using spectral entropy to compute clusters, noise clusters, mixed clusters, and information clusters can be quickly distinguished. The mixed clusters are denoised by wavelet threshold and the signals are reconstructed to finally obtain the denoised useful signal. The experimental results show that the ISGMD-WT algorithm has outstanding denoising capability, the highest SNR, and the smallest RMSE. It can effectively denoise the hydrophone signals to obtain complete information from the underwater acoustic signals.

## Figures and Tables

**Table 1:** Evaluation indexes for different denoising algorithms.

Index	Original Signal	WT	EMD-WT	VMD-WT	ISGMD-WT
**SNR**	5.63	6.45	8.45	10.09	21.34
**RMSE**	1.8716	1.6826	1.3364	1.1067	0.3031

**Table 2:** Denoising evaluation indexes of the four algorithms under different noise decibels.

White Noise	Index	Original Signal	WT	EMD-WT	VMD-WT	ISGMD-WT
0 db	**SNR**	−2.64	0.96	4.90	8.58	9.23
	**RMSE**	1.9360	1.7912	1.1375	0.9415	0.6909
5 db	**SNR**	−1.28	6.04	5.08	10.19	13.43
	**RMSE**	1.1888	0.9981	1.1139	0.6380	0.4261
10 db	**SNR**	−0.33	8.10	8.64	9.37	17.87
	**RMSE**	0.7059	0.7867	0.7393	0.6800	0.2557

**Table 3:** Evaluation indexes for denoising hydrophone signal.

Index	WT	EMD-WT	VMD-WT	ISGMD-WT
**SNR**	23.96	29.31	36.07	42.18
**RMSE**	0.008401	0.005637	0.001555	0.001041
